# Screening and Identification of Differentially Expressed Genes Expressed among Left and Right Colon Adenocarcinoma

**DOI:** 10.1155/2020/8465068

**Published:** 2020-01-21

**Authors:** Jing Han, Xue Zhang, Yang Yang, Li Feng, Gui-Ying Wang, Nan Zhang

**Affiliations:** ^1^Department of Medical Oncology, The Fourth Hospital of Hebei Medical University, Shijiazhuang, Hebei 050000, China; ^2^Second Department of Surgery, The Fourth Hospital of Hebei Medical University, Shijiazhuang, Hebei 050000, China; ^3^Department of Thoracic Surgery, The Fourth Hospital of Hebei Medical University, Shijiazhuang, Hebei 050000, China

## Abstract

**Purpose:**

Colon adenocarcinoma (COAD) is the third most common malignancy globally and is further categorized as left colon adenocarcinoma (LCOAD) or right colon adenocarcinoma (RCOAD) depending on the location of the primary tumor. The therapeutic outcome and long-term prognosis for patients with COAD are less than satisfactory, and this may be associated with tumor location. Therefore, it is important to investigate the genetic differences in COAD at different sites. *Patients and Methods*. Public data associated with COAD were downloaded from the Gene Expression Omnibus (GEO) database. Differentially expressed genes (DEGs) were identified using R software (version 3.5.3), and functional annotation of DEGs was performed using Gene Ontology (GO) and Kyoto Encyclopedia of Genes and Genomes (KEGG) analyses. A protein-protein interaction network was constructed, hub genes were identified and analyzed, and data mining using Gene Expression Profiling Interactive Analysis (GEPIA) was conducted.

**Results:**

A total of 286 DEGs were identified between LCOAD and RCOAD. Additionally, 10 hub genes associated with COAD at different locations were screened, namely, CDKN2A, IGF1R, MDM2, SMAD3, SLC2A1, GRM5, PLCB4, FGFR1, UBE2V2, and TNFRSF10B. The expression of cyclin-dependent kinase inhibitor 2A (CDKN2A) and solute carrier family 2 member 1 (SLC2A1) was significantly associated with pathological stage (*P* < 0.05). COAD patients with high expression levels of CDKN2A exhibited poorer overall survival (OS) times than those with low expression levels (*P* < 0.05).

**Conclusion:**

CDKN2A expression was significantly different between LCOAD and RCOAD and was closely related to the prognosis of COAD. It is of great value for further understanding of the pathogenesis of LCOAD and RCOAD.

## 1. Introduction

Colon adenocarcinoma (COAD) is the third most common malignancy worldwide, accounting for 10.0% of all new cancer cases, and is one of the leading causes of cancer-associated mortality [1]. The incidence of COAD has increased year on year and is closely associated with genetic, environmental, and dietary changes, as well as colonic mucosal hyperplasia and the canceration of adenomatous polyps [2]. With the development of targeted therapy, great progress has been made in the treatment of COAD, but the therapeutic outcome and long-term prognosis of patients remain unsatisfactory. It has been suggested that this may be associated with the location of the tumor; thus, the investigation of differences in the incidence of COAD at different sites is particularly important.

Based on tumor location, COAD includes at least two types [3], left colon adenocarcinoma (LCOAD) and right colon adenocarcinoma (RCOAD). LCOAD refers to tumors from the splenic flexure of the colon to the sigmoid colon, and RCOAD refers to tumors between the ileocecal region and the transverse colon [4]. In addition to their different origins, LCOAD and RCOAD also have different clinical manifestations, histological types, molecular characteristics, prognoses, modes of metastasis, and treatment options [3], which are reflected in the following aspects.

In terms of clinical manifestation, hematochezia and changes in bowel habits are more frequently associated with LCOAD, while iron-deficiency anemia caused by occult blood loss is more common in patients with RCOAD [5]. The data showed that RCOAD patients were more likely to be female, of older age, with larger tumor diameters, poor differentiation, later Tumor-Node-Metastasis stages, and shorter survival times compared with LCOAD patients [6, 7]. In the past 30 years, the incidence of RCOAD has risen, and its incidence is now reportedly higher than that of LCOAD [8]. From a molecular perspective, RCOAD and LCOAD are two separate entities. The fundamental reason for the obvious difference between RCOAD and LCOAD lies in the difference of molecular typing. For example, in the RCOAD, there are high mutations of genes, methylation, BRAF (B-Raf Proto-Oncogene, Serine/Threonine Kinase) mutation, serrated pathway, and inflammatory. And the prognosis of the RCOAD is poor [9]. However, in the LCOAD, there exist chromosomal instability, amplification of EGFR1 (Epidermal Growth Factor Receptor 1) and EGFR2 (Epidermal Growth Factor Receptor 2), EGF (Epidermal Growth Factor) signal transduction, and Wnt signal transduction. 13% of the LROAD with BRAF mutation has a poor prognosis, while 87% without BRAF mutation will have a good prognosis [9]. RCOAD is related to KRas and Serine/threonine-protein kinase B-raf (BRAF) mutations of defect mismatch repair genes and microRNA-31, while LCOAD is closely associated with chromosome instability, p53, NRas, and microRNA-146a, microRNA-147b, and microRNA-1288 [10]. However, Gao et al. [11] showed no significant difference in the expression levels of MLH1, MSH2, MSH6, PMS2, *β*-tubulin III, p53, Ki67, topoisomerase Ii*α*, and BRAF gene mutations between the two types of COAD. A number of studies have reported significant differences in p53 gene mutation and protein expression between RCOAD and LCOAD [12–14], while another study has shown no significant correlation between p53 protein expression and tumor location [15]. Therefore, it is significantly necessary to identify the differentially expressed genes between RCOAD and LCOAD.

Bioinformatics is a comprehensive field that integrates biology, computer science, and mathematics [16]. With the development of sequencing technology, bioinformatics data has rapidly accumulated and is widely used in medicine and drug development. Concurrently, much gene expression profile data have been generated [17], and efficient data mining has become a bioinformatics research hotspot. The development of bioinformatics also provided a novel approach for the discovery and identification of differentially expressed genes (DEGs) between LCOAD and RCOAD [18].

In the present study, COAD gene chip data from the Gene Expression Omnibus (GEO) were analyzed to identify DEGs and hub genes between LCOAD and RCOAD, construct an interaction network of DEGs, and conduct Gene Ontology (GO) and Kyoto Encyclopedia of Genes and Genomes (KEGG) analyses between these genes. These DEGs and hub genes may provide new ideas to study the differences between LCOAD and RCOAD and the subsequent development of targeted therapy.

## 2. Materials and Methods

### 2.1. Access to Public Data

The GEO (http://www.ncbi.nlm.nih.gov/geo) is an open-source platform for the storage of genetic data [19]. Two expression profiling datasets (GSE81558 (GPL15207 platform) and GSE75317 (GPL570 platform)) were, respectively, downloaded from the GEO database. The GSE81558 dataset includes 9 normal colorectal tissues, 19 liver tissues from colorectal liver metastasis patients, 12 rectum tissues from primary colorectal tumor patients, 9 left colon tissues from primary colorectal tumor patients, and 2 right colon tissues from primary colorectal tumor patients. This study mainly aimed to identify the differentially expressed genes between left colorectal tumors and right colorectal tumors. Therefore, we chose only 9 LCOAD and 2 RCOAD samples from the GSE81558 dataset based on the source type. Similarly, 33 LCOAD samples and 26 RCOAD samples were selected from GSE75315 (GPL570 platform).

### 2.2. DEGs Identified Using R Software

R software (version 3.5.3) is used to distinguish DEGs between LCOAD and RCOAD tissue samples. If one probe set does not contain the homologous gene, or if one gene has numerous probe sets, the data is removed. *P* < 0.05 is considered to indicate a statistically significant difference. The DEGs are presented as volcano plots, generated using SangerBox software (http://sangerbox.com/), and Venn diagrams were constructed using FunRich software (http://www.funrich.org).

### 2.3. Functional Annotation of DEGs Using KEGG and GO Pathway Enrichment Analyses

The Database for Annotation, Visualization, and Integrated Discovery (DAVID) (https://david.ncifcrf.gov/home.jsp; version 6.8) is an online suite of analysis tools with an integrated discovery and annotation function [20]. The GO resource is widely used in bioinformatics and covers three aspects of biology, including biological process (BP), cellular component (CC), and molecular function (MF) [21]. KEGG (https://www.kegg.jp/) is one of the most commonly used biological information databases worldwide [22]. DAVID was used to perform GO and KEGG analyses of DEGs, and *P* < 0.05 was considered to indicate a statistically significant difference.

### 2.4. Construction of a Protein-Protein Interaction (PPI) Network

Search Tool for the Retrieval of Interacting Genes (http://string.embl.de/), an open-source online tool, was used to construct a PPI network of the identified DEGs, and Cytoscape visualization software version 3.6.1 [23] was used to present the network [24]. A confidence score >0.4 was considered as the criterion of judgment, which may filter out the critical module.

### 2.5. Identification and Analysis of Hub Genes

Functional annotation of the genes was performed using KEGG and GO analyses in DAVID. A single coexpression network was constructed using cBioPortal (http://www.cbioportal.org) [25]. The Biological Networks Gene Oncology tool (BiNGO) version 3.0.3, one plug-in of the Cytoscape, was used to analyze and visualize the BPs and MFs of each hub gene [26]. OmicShare (http://www.omicshare.com/tools), an open data analysis platform, was subsequently used to perform clustering analysis of these genes.

### 2.6. Data Mining Using Gene Expression Profiling Interactive Analysis (GEPIA)

The correlations between gene expression and pathological stage were ascertained using GEPIA (http://gepia.cancer-pku.cn/), a newly developed interactive web server for analyzing the gene expression data of large consortium projects such as The Cancer Genome Atlas and the Genotype Tissue Expression project [27]. Correlations between pathological stage, overall survival (OS), and the expression of hub genes in COAD were also identified using GEPIA. The correlation between SLC2A1 and GLUT1 expression was tested by GEPIA.

### 2.7. RT-qPCR Assay

A total of 8 participates were recruited, including 4 LCOAD and 4 RCOAD samples. After surgery, 4 LCOAD samples from LCOAD patients and 4 RCOAD samples from control individuals were obtained. The research conformed to the Declaration of Helsinki and was authorized by the Human Ethics and Research Ethics Committees of the Fourth Hospital of Hebei Medical University. An informed consent was obtained from all participants.

Total RNA was extracted from 4 LCOAD samples and 4 RCOAD samples by the RNAiso Plus (Trizol) kit (Thermofisher, Massachusetts, America) and reverse transcribed to cDNA. RT-qPCR was performed using a Light Cycler® 4800 System with specific primers for the ten hub genes.  Table 1 presents the primer sequences used in the experiments. The RQ values (2^−ΔΔ*Ct*^, where *Ct* is the threshold cycle) of each sample were calculated and are presented as fold change in gene expression relative to the control group. GAPDH was used as an endogenous control.

### 2.8. Overall Survival Analysis of the LCOAD and RCOAD

The present study recruited a total of 106 LCOAD and 106 RCOAD patients from the Fourth Hospital of Hebei Medical University. Clinical and histopathological characteristics and follow-up and survival information were available for all patients and were collected retrospectively from medical records. Patients who are aged 30 to 100 years old, are histologically confirmed as colorectal adenocarcinoma [28], do not receive tumor treatment, and have no history of surgery [29] will be screened for inclusion criteria. Exclusion criteria included the following: age <30 years old or >100 years old, combined with other malignant tumors, operation time more than 1 month after the last examination, and severe heart disease. The expression level of CDKN2A in LCOAD or RCOAD patients was measured by RT-qPCR. In this clinical study, we followed up the patients for 210 months. The endpoint of the study was death from colon adenocarcinoma. This trial and the informed consent forms have been reviewed and approved by the Ethics Review Committee of Fourth Hospital of Hebei Medical University, and the approval number is 2017MEC115. The Kaplan–Meier method was performed to analyze the overall survival. All statistical analyses were conducted using SPSS software (version 21.0), and *P* < 0.05 was considered statistically significant.

## 3. Results

### 3.1. Screening of DEGs between LCOAD and RCOAD

**Figure 1:**
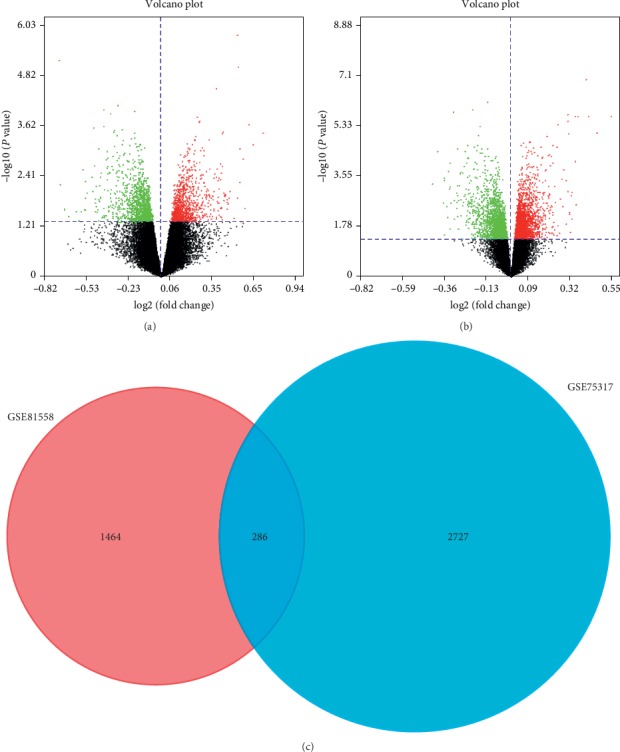
Identification of differentially expressed genes. Volcano plots present the difference between LCOAD and RCOAD samples of the (a) GSE81558 and (b) GSE75317 datasets. (c) Venn diagram identifying 286 common genes between the two datasets.

In the GSE81558 dataset, we chose nine LCOAD and two RCOAD samples into this research. And in the GSE75317 dataset, we chose 33 LCOAD and 26 RCOAD samples into this research. Following the analysis of the GSE81558 and GSE75317 datasets, respectively, the differences between LCOAD and RCOAD tissues in GSE81558 and GSE75317 were presented as volcano plots as shown in Figures 1(a) and 1(b), respectively. A Venn diagram revealed 286 common DEGs between the two datasets (Figure 1(c)).

### 3.2. Functional Annotation for DEGs Using KEGG and GO Analyses

The results of GO analysis revealed that variations in the BP were predominantly enriched in protein complex assembly, sialylation, oligosaccharide metabolic process, peptidyl-tyrosine, phosphorylation, and apoptotic process. Changes in CC were primarily enriched in intracellular, cell-cell junction, peroxisomal matrix, cytosol, and postsynaptic density. Variations in MF were enriched in metal ion binding, sialyltransferase activity, transcription factor activity, sequence-specific DNA binding, nucleic acid binding, and protein binding (Table 2). KEGG analysis demonstrated that DEGs were largely enriched in transcriptional misregulation in cancer, pathways in cancer, and peroxisome (Table 2).

### 3.3. Construction of the PPI Network

**Figure 2:**
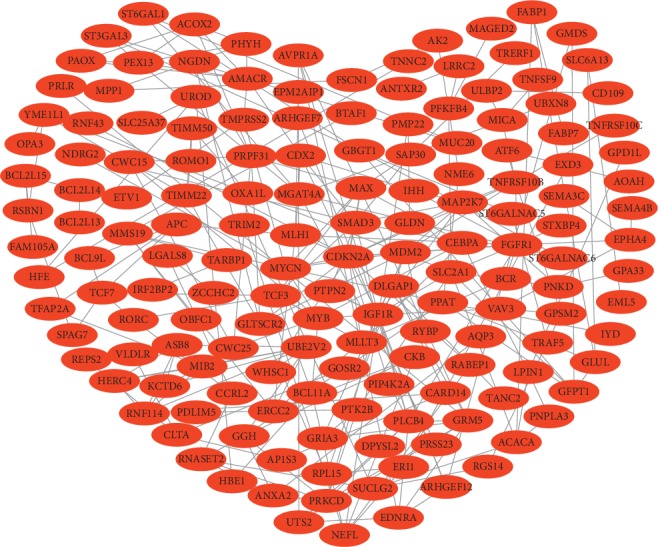
Protein-protein interaction network of differentially expressed genes, consisting of 264 edges and 159 nodes.

The construction of a PPI network revealed 264 edges and 159 nodes in the PPI network (PPI enrichment; *P*=0.0112; Figure 2). The network possessed significantly more interactions than expected, highlighting a greater number of interactions between DEGs than expected for a random set of proteins of a similar size from the same genome. Such enrichment indicates that the identified proteins are at least partially associated.

### 3.4. Hub Gene Selection and Functional Annotation

**Figure 3:**
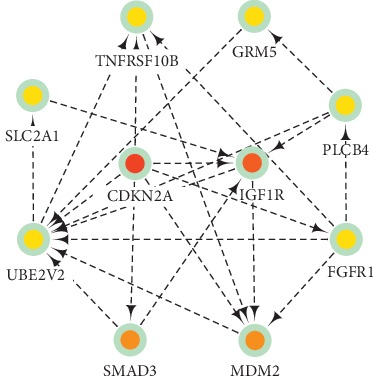
Hub genes identified within the protein-protein interaction network.

The following 10 hub genes were identified using Cytoscape, and KEGG and GO analyses were conducted using DAVID: CDKN2A, IGF1R, MDM2, SMAD3, SLC2A1, GRM5, PLCB4, FGFR1, UBE2V2, and TNFRSF10B (Figure 3). The results of GO analysis showed that variations in the BP were largely enriched in the activation of cysteine-type endopeptidase activity involved in the apoptotic process, activation of cysteine-type endopeptidase activity involved in the apoptotic signaling pathway, protein destabilization, protein K63-linked ubiquitination, and immune response. Variations in the CC were predominantly enriched in receptor complex, integral component of plasma membrane, plasma membrane, and cytosol, whereas those in the MF were enriched in identical protein binding, SUMO transferase activity, ubiquitin protein ligase binding, protein binding, and p53 binding. KEGG pathway analysis revealed that the hub genes were mainly enriched in pathways in cancer, adherens junction, cell cycle, FoxO signaling pathway, and proteoglycans in cancer (Table 3). Summaries of the functions of all hub genes are presented in Table 4.

### 3.5. Analysis of Hub Genes

**Figure 4:**
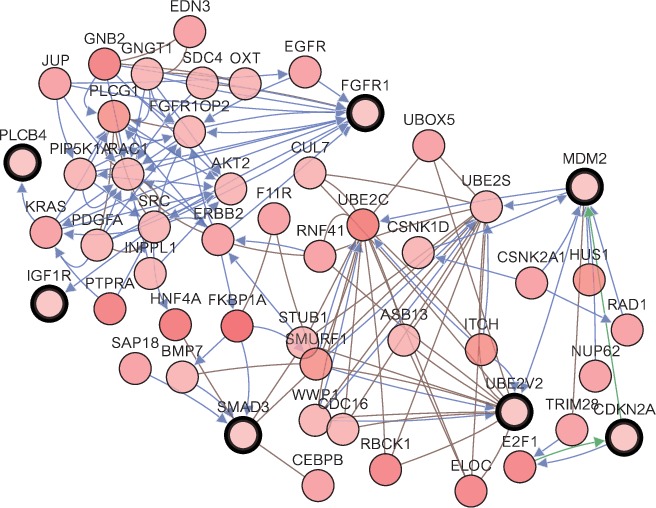
Coexpression network of hub genes obtained using cBioPortal.

**Figure 5:**
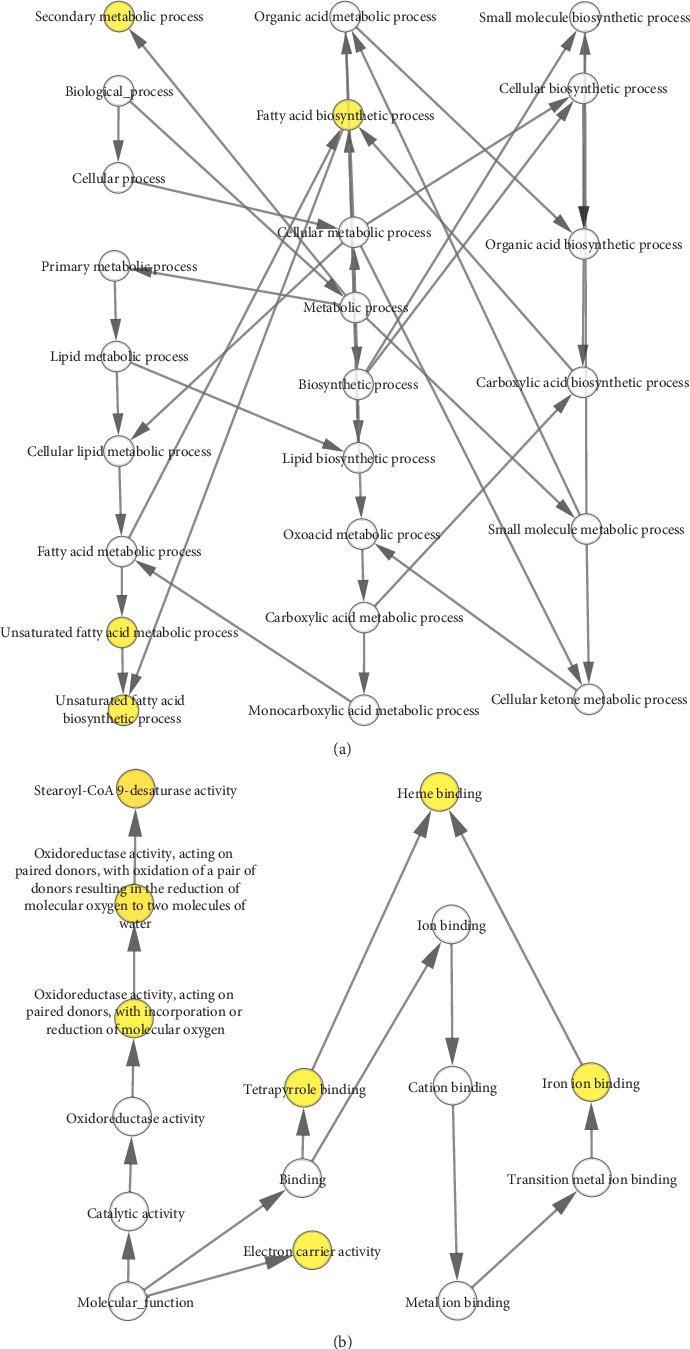
(a) Biological process and (b) molecular function analysis of the identified hub genes using the Biological Networks Gene Oncology tool.

**Figure 6:**
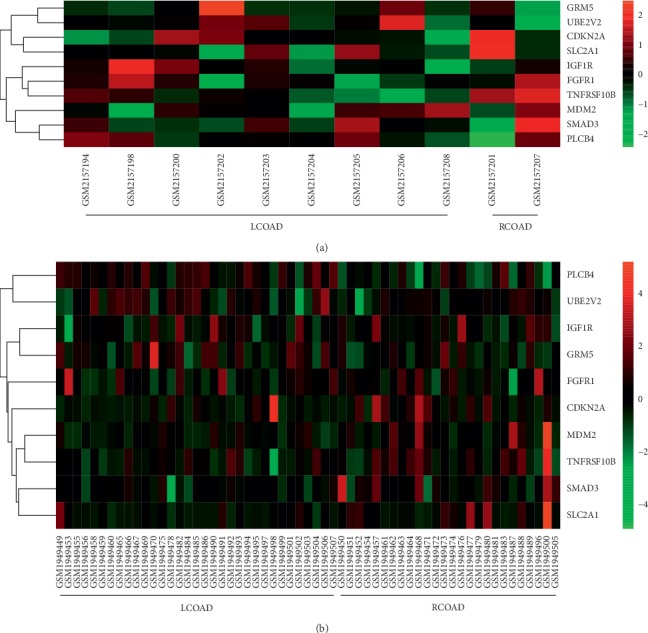
Hierarchical clustering. Differentiation between RCOAD and LCOAD samples in the (a) GSE81558 and (b) GSE75317 datasets using the identified hub genes. The color represents the expression level of each gene (green, low expression; black, medium expression; and red, high expression).

A coexpression network of the hub genes was constructed using cBioPortal. Among these genes, CDKN2A, UBE2V2, MDM2, SMAD3, FGFR1, IGF1R, and PLCB4 exhibited the highest node scores, suggesting that they may possess pivotal functions for distinguishing between LCOAD and RCOAD (Figure 4). Using the BiNGO tool, biological process analysis of the hub genes is illustrated in Figure 5(a), and molecular function analyses of the hub genes are presented in Figure 5(b). Hierarchical clustering revealed that the hub genes were able to differentiate between the LCOAD and RCOAD samples (Figure 6). Within the GSE81558 dataset, when compared with LCOAD, the expression of GRM5 and UBE2V2 was downregulated, and that of CDKN2A, SLC2A1, IGF1R, FGFR1, TNFRSF10B, MDM2, SMAD3, and PLCB4 was upregulated in RCOAD (Figure 6(a)). In the GSE75317 dataset, when compared with LCOAD, expression levels of PLCB4 and UBE2V2 were downregulated, while those of CDKN2A, MDM2, TNFRSF10B, SMAD3, and SLC2A1 were upregulated in RCOAD (Figure 6(b)).

### 3.6. RT-qPCR Analysis Validation of Hub Genes

**Figure 7:**
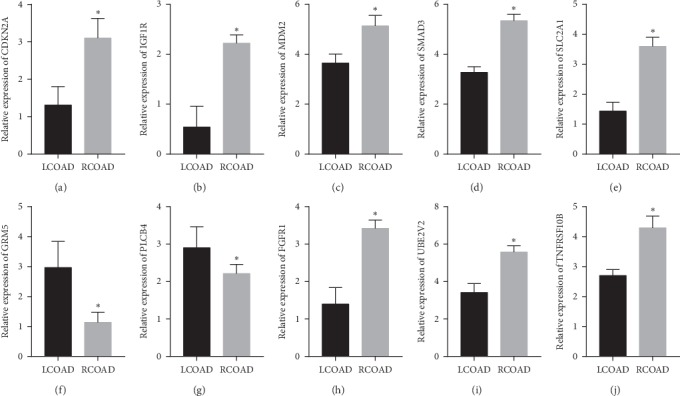
Relative expression of hub genes between LCOAD and RCOAD by RT-qPCR analysis. (a) CDKN2A, (b) IGF1R, (c) MDM2, (d) SMAD3, (e) SLC2A1, (f) GRM5, (g) PLCB4, (h) FGFR1, (i) UBE2V2, and (j) TNFRSF10B. ^*∗*^*P* < 0.05.

As presented in the result, GRM5 and PLCB4 were markedly downregulated in RCOAD samples, when compared with the LCOAD. The relative expression levels of CDKN2A, IGF1R, MDM2, SMAD3, SLC2A1, FGFR1, UBE2V2, and TNFRSF10B were significantly higher in RCOAD samples, compared with the LCOAD groups (Figure 7). It should be noted that CDKN2A, MDM2, SMAD3, SLC2A1, and TNFRSF10B were consistent with the above results.

### 3.7. The Relationship between Pathological Stage, OS, and the Expression of Hub Genes

**Figure 8:**
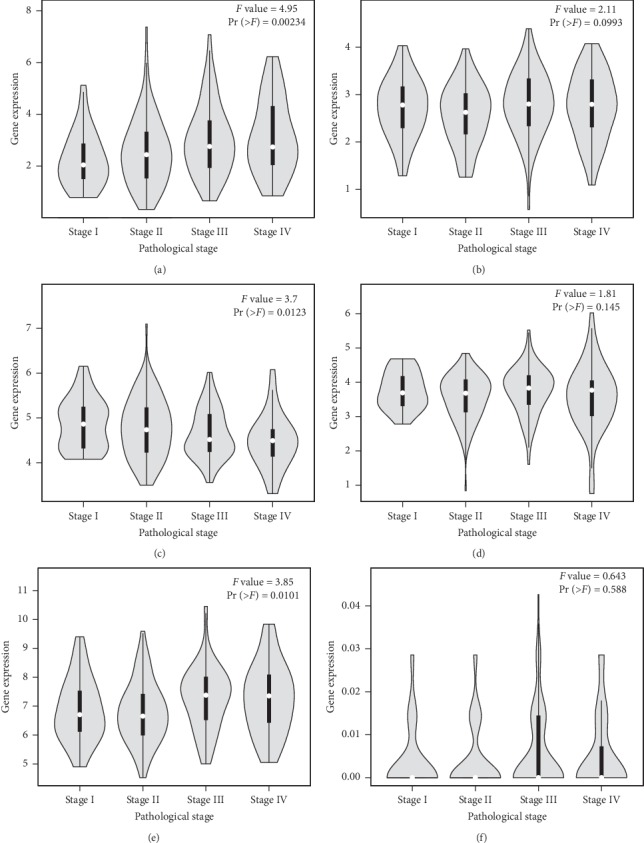
Association between pathological stage and the expression levels of (a) CDKN2A, (b) IGF1R, (c) MDM2, (d) SMAD3, (e) SLC2A1, and (f) GRM5.

**Figure 9:**
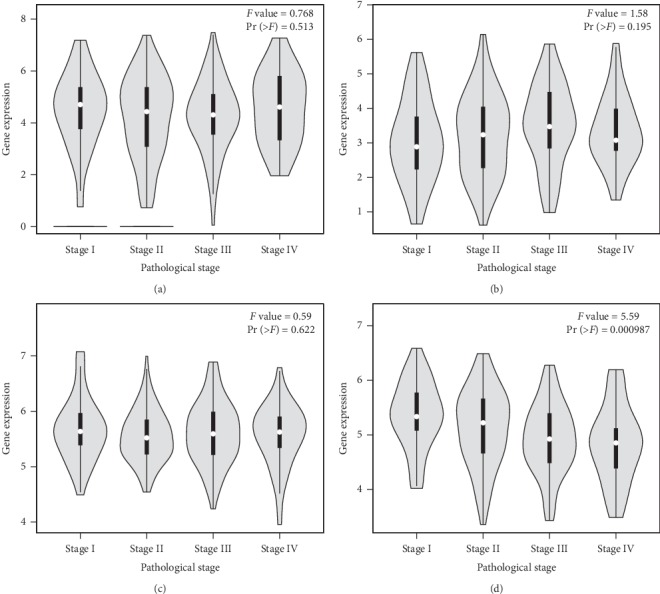
Association between pathological stage and the expression levels of (a) PLCB4, (b) FGFR1, (c) UBE2V2, and (d) TNFRSF10B.

**Figure 10:**
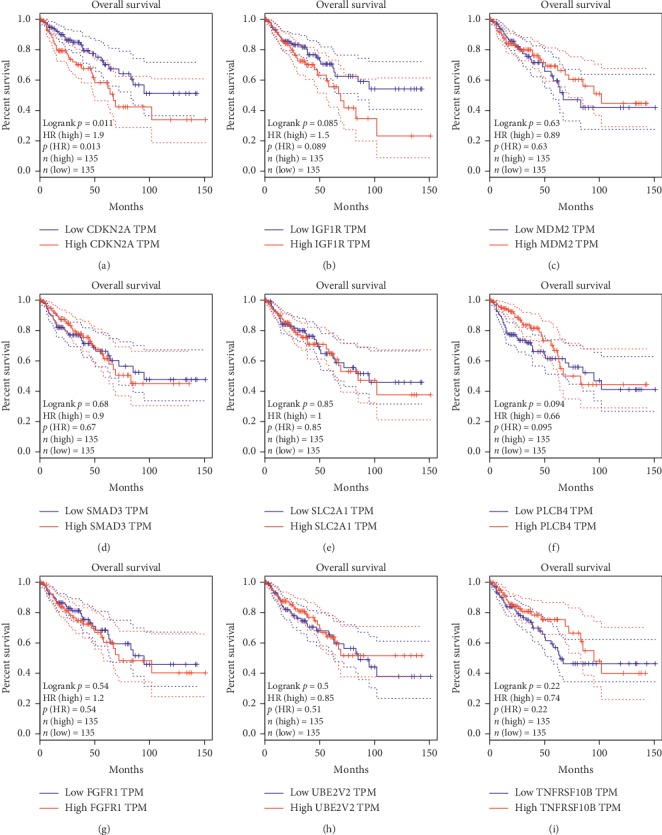
Kaplan–Meier overall survival analysis using Gene Expression Profiling Interactive Analysis. (a) CDKN2A, (b) IGF1R, (c) MDM2, (d) SMAD3, (e) SLC2A1, (f) PLCB4, (g) FGFR1, (h) UBE2V2, and (i) TNFRSF10B. The expression level of CDKN2A is closely correlated with the prognosis of COAD patients (*P* < 0.05).

GEPIA analysis showed that the expression of CDKN2A, MDM2, SLC2A1, and TNFRSF10B was significantly associated with pathological stage (*P* < 0.05; Figures 8(a), 8(c), 8(d), and 8(e)), while the expression of IGF1R, SMAD3, GRM5, PLCB4, FGFR1, and UBE2V2 was not (Figures 8(b), 8(d), 8(f) and 9(a)–9(c)). The pathological stage of COAD was positively related to the expression of CDKN2A and SLC2A1 and negatively related to the expression of MDM2 and TNFRSF10B. Kaplan–Meier analysis using GEPIA revealed that COAD patients with high expression levels of CDKN2A had poorer overall survival times than those with low expression levels (*P* < 0.05; Figure 10(a)); there was no statistically significant effect on OS associated with the expression of IGF1R, MDM2, SMAD3, SLC2A1, GRM5, PLCB4, FGFR1, UBE2V2, or TNFRSF10B (*P* > 0.05; Figures 10(b)–10(i)). Therefore, the other nine genes are not related to the prognosis. After the analysis by GEPIA, there exists a positive correlation between SLC2A1 and GLUT1 expression levels (*R* = 1, *P* < 0.001).

### 3.8. High Expressions of CDKN2A in Patients with LCOAD or RCOAD Were Independent Prognostic Factors for the Poor Overall Survival

**Figure 11:**
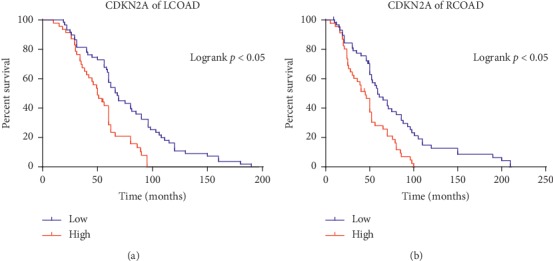
The Kaplan–Meier OS curves of the LCOAD and RCOAD patients with the low/high expression of CDKN2A. (a) High expression of CDKN2A was a predictor of a shorter OS in the LCOAD patients (*P* < 0.05). (b) High expression of CDKN2A was a predictor of a shorter OS in the RCOAD patients (*P* < 0.05).

The demographic data and the expression status of CDKN2A were summarized in Table 5. The Kaplan–Meier OS curves were presented in Figure 11. High expression of CDKN2A was a predictor of a shorter OS in the LCOAD patients (Figure 11(a)) and RCOAD patients (Figure 11(b)).

## 4. Discussion

With global changes in diet and lifestyle, COAD-associated morbidity and mortality have increased, making it one of the primary malignant tumors threatening human health. There is no consensus on the relationship between tumor location and the pathological stage and prognosis of COAD. A meta-analysis [30] of 66 studies that analyzed the OS data of 1.43 million COAD patients showed a 19% reduction in mortality among patients with LCOAD, compared with those with RCOAD; this suggested that the location of the primary tumor serves a key role in determining the prognosis of colon adenocarcinoma. However, Weiss et al. [7] found no significant difference in the 5-year OS rates between patients with left and right COAD, following the adjustment for various prognostic factors. In addition, numerous studies have reported differences in the molecular mechanisms of COAD at different locations [10, 31, 32], but it was not clear whether these molecular differences could be translated into clinically meaningful changes in pathological stage and prognosis. Therefore, pathological stage and prognosis may serve important roles in investigating the relationship between the molecular mechanisms of the occurrence and development of COAD at different locations, facilitating the screening, diagnosis, and targeted treatment of patients with COAD [33].

Bioinformatics is the computational science of understanding biological and genetic information for the purpose of expanding the use of biological and medical data [34]. The units of bioinformatics research are DNA, RNA, and protein molecules, which can be reliably utilized for the identification and investigation of DEGs [35, 36]. COAD results from the interaction of multiple genes and the bioinformatic application of gene expression profiles provide the possibility of studying the pathogenesis of COAD at different locations. Furthermore, the biological analysis of gene chip data is another important advancement for data mining [37].

In the present study, bioinformatics technology was used to analyze two datasets (GSE81558 and GSE75317), in which a total of 286 DEGs were identified. GO enrichment analysis, KEGG signal pathway analysis, and PPI network analysis were also performed with these DEGs, and the following ten hub genes associated with COAD at different locations were identified by the cytoHubba when the degree ≥10, one plug-in of Cytoscape software: CDKN2A, IGF1R, MDM2, SMAD3, SLC2A1, GRM5, PLCB4, FGFR1, UBE2V2, and TNFRSF10B. Among these genes, the expression of CDKN2A and SLC2A1 was upregulated in RCOAD, compared with LCOAD. GEPIA showed that the expression of CDKN2A was significantly associated with pathological stage (*P* < 0.05). With the increase in CDKN2A expression levels, the pathological stage of COAD also increased (*P* < 0.05). Kaplan–Meier curve analysis using GEPIA revealed that COAD patients with high expression levels of CDKN2A had poorer OS times than those with low expression levels (*P* < 0.05).

Cyclin-Dependent Kinase Inhibitor 2A (CDKN2A) is an important tumor suppressor gene belonging to the family of cyclin-dependent kinase inhibitor genes, which serves a regulatory role in cell proliferation and apoptosis [38]. The pathways associated with CDKN2A are signaling and apoptosis modulation. CDKN2A codes for two cyclic inhibitory proteins, p16INK4a and p14ARF. Furthermore, through the p16ink4a-cdk4 (and CDK6)-prb and p14arf-mdm2-p53 pathways, it serves a role in cell cycle regulation. CDKN2A is able to induce cell cycle arrest at the G_1_ and G_2_ phases and thus has a tumor-inhibitory effect [39]. CDKN2A binds the proto-oncogene MDM2 and blocks its karyoplasmic shuttling by sequestrating MDM2 in the nucleolus. In addition, MDM2-induced degradation of p53 was blocked, enhancing p53-dependent activation and subsequent apoptosis, thereby inhibiting the carcinogenic effect of MDM2 [40]. Additionally, CDKN2A is able to bind BCL6, downregulating bcl6-induced transcriptional inhibition; it can also bind E2F1 and MYC, blocking the transcriptional activation activity of E2F1. However, no effect on MYC-associated transcriptional inhibition has been reported.

CDKN2A mutation has been demonstrated as an important event in a number of tumor types, including pancreatic cancer [41] and gastric cancer. Therefore, the development of cancer is often accompanied by CDKN2A mutations; the loss of its anticancer function may promote the neoplastic transformation of cells, subsequently inducing proliferation, invasion, and metastasis [42]. In the present study, it was speculated that CDKN2A may be mutated in COAD, the pathological stage of COAD was positively related to the expression of CDKN2A, and the mutated protein may promote the abnormal proliferation and differentiation of colonic glandular epithelial cells. The results indicated that the expression level of CDKN2A in RCOAD was higher than that in LCOAD and that this is positively correlated with the pathological stage of patients with COAD. Survival analysis also revealed that when CDKN2A was highly expressed, the OS rate of patients with COAD was low and the prognosis was poor. This suggested a possible reason (and research direction) for the hypothesis that, at the molecular level, patients with RCOAD possess a higher pathological stage and poorer prognosis than those with LCOAD.

However, there are still some shortcomings to the present study. The sample size of only two datasets was relatively small. In the result of hierarchical clustering data, PLCB4 expression was upregulated in RCOAD as compared to LCOAD using the GSE81558 dataset while PLCB4 expression was downregulated using the GSE75317 dataset. We think that the reasons causing this situation are small sample sizes and individual differences. Currently, there are some research studies about the difference between RCOAD and LCOAD in genomics. Based on the previous studies, our study creatively identified critical differentially expressed genes between LCOAD and RCOAD through the bioinformatics method and further verified them in clinical samples. We found that CDKN2A is expected to be a key target for the pathogenesis and treatment of LCOAD and RCOAD. Meanwhile, a large number of clinical samples and animal experiments would provide more comprehensive verification and a deeper understanding of the different molecular mechanisms, clinical pathological staging, and survival differences between RCOAD and LCOAD.

## 5. Conclusion

We studied the gene difference between LCOAD and RCOAD by bioinformatics and verified the result by molecular biology, in an attempt to deeply understand the pathogenesis of COAD and expand the thinking for the discovery of new therapeutic targets. Our study identified 286 differentially expressed genes and 10 hub genes, with a focus on verifying the differential expression and prognostic value of CDKN2A. The expression of CDKN2A is upregulated in the RCOAD and is downregulated in the LCOAD. The higher the expression of CDKN2A is, the poorer the pathological stage and overall survival are. Therefore, the prognosis of LCOAD is better than RCOAD. The present study has provided a reference point for the in-depth study of COAD-associated genes, the discovery of molecular markers at different locations, and the biological processes in which they are involved.

## Figures and Tables

**Table 1:** Primers and their sequences for PCR analysis.

Primer	Sequence (5′–3′)
CDKN2A-hF	ATATAGCTTCAAAAAGCAAAGGC
CDKN2A-hR	TTAAAATCAAATCCAGCAACAGG
IGF1R-hF	GAAGTTGAGAAGGAATGAAGACA
IGF1R-hR	AATCACCCAAGAAAACAAGACAG
MDM2-hF	CCAAGGGGGGTAGTAAAGGGTAT
MDM2-hR	TAGAAGGCAAGGAAGAAAGGAGT
SMAD3-hF	CACTCGGGAATGGGAAAAATGAA
SMAD3-hR	AAAAAATAGCCAGGCGTGGTAGC
SLC2A1-hF	GCATGGGTGATGTGTGGTTTGAA
SLC2A1-hR	AGGGTATCCTCTCCTGGTTTTAG
GRM5-hF	AGGACAGTAAACCAGGAAGCAGG
GRM5-hR	GAGGTAATTGAATCATAGGGGCG
PLCB4-hF	TGCTTTAATTTTATTATACCCCC
PLCB4-hR	AAGTCTCAGTCAATCCAGTCCTC
FGFR1-hF	GCCAGAGCAAGTGTGGGTTTTAT
FGFR1-hR	GATGCGTGTGATTCGGAGAGGGT
UBE2V2-hF	AGGTTCACTCCTCATTCTTTTTT
UBE2V2-hR	TTTTCCCTATTTGATGTTTCTGT
TNFRSF10B-hF	AATATACGCAGGATTTGAAGACG
TNFRSF10B-hR	ACATTAAAAAAGGTGAGAAGGGG

**Table 2:** GO and KEGG pathway enrichment analyses of DEGs between left and right COAD.

Term	Description	Count in gene set	*P* value
GO:0006461	Protein complex assembly	8	0.002
GO:0097503	Sialylation	4	0.003
GO:0009311	Oligosaccharide metabolic process	4	0.006
GO:0018108	Peptidyl-tyrosine phosphorylation	8	0.009
GO:0006915	Apoptotic process	17	0.016
GO:0005622	Intracellular	35	9.01*E* − 04
GO:0005911	Cell-cell junction	9	0.004
GO:0005782	Peroxisomal matrix	5	0.004
GO:0005829	Cytosol	63	0.017
GO:0014069	Postsynaptic density	8	0.018
GO:0046872	Metal ion binding	48	0.002
GO:0008373	Sialyltransferase activity	4	0.003
GO:0003700	Transcription factor activity, sequence-specific DNA binding	26	0.005
GO:0003676	Nucleic acid binding	26	0.007
GO:0005515	Protein binding	152	0.008
hsa05202	Transcriptional misregulation in cancer	11	4.20 − 04
hsa05200	Pathways in cancer	16	0.002
hsa04146	Peroxisome	5	0.048

GO: Gene Ontology; KEGG: Kyoto Encyclopedia of Genes and Genomes; DEGs: differentially expressed genes. COAD: colon adenocarcinoma.

**Table 3:** GO and KEGG pathway enrichment analyses of hub genes between left and right COAD.

Term	Description	Count in gene set	*P* value
GO:0006919	Activation of cysteine-type endopeptidase activity involved in apoptotic process	3	8.50*E *−* *04
GO:0097296	Activation of cysteine-type endopeptidase activity involved in apoptotic signaling pathway	2	0.007
GO:0031648	Protein destabilization	2	0.019
GO:0070534	Protein K63-linked ubiquitination	2	0.020
GO:0006955	Immune response	3	0.020
GO:0043235	Receptor complex	3	0.002
GO:0005887	Integral component of plasma membrane	5	0.003
GO:0005886	Plasma membrane	7	0.006
GO:0005829	Cytosol	6	0.013
GO:0042802	Identical protein binding	5	4.05*E *−* *04
GO:0019789	SUMO transferase activity	2	0.009
GO:0031625	Ubiquitin protein ligase binding	3	0.010
GO:0005515	Protein binding	9	0.026
GO:d0002039	p53 binding	2	0.035
hsa05200	Pathways in cancer	7	8.51*E *−* *07
hsa04520	Adherens junction	3	0.003
hsa04110	Cell cycle	3	0.008
hsa04068	FoxO signaling pathway	3	0.010
hsa05205	Proteoglycans in cancer	3	0.021

GO: Gene Ontology; KEGG: Kyoto Encyclopaedia of Genes and Genomes; DEGs: differentially expressed genes; COAD: colon adenocarcinoma.

**Table 4:** Summaries for the function of 10 hub genes.

No.	Gene symbol	Full name	Function
1	CDKN2A	Cyclin-dependent kinase inhibitor 2A	Capable of inducing cell cycle arrest in G1 and G2 phases. Acts as a tumor suppressor. Acts as a negative regulator of the proliferation of normal cells by interacting strongly with CDK4 and CDK6
2	IGF1R	Insulin-like growth factor 1 receptor	The activated IGF1R is involved in cell growth and survival control. IGF1R is crucial for tumor transformation and survival of malignant cell
3	MDM2	MDM2 proto-oncogene	Inhibits p53/TP53- and p73/TP73-mediated cell cycle arrest and apoptosis by binding its transcriptional activation domain. Inhibits DAXX-mediated apoptosis by inducing its ubiquitination and degradation
4	SMAD3	SMAD family member 3	Receptor-regulated SMAD (R-SMAD) that is an intracellular signal transducer and transcriptional modulator activated by TGF-beta (transforming growth factor) and activin type 1 receptor kinases
5	SLC2A1	Solute carrier family 2 member 1	Facilitative glucose transporter. This isoform may be responsible for constitutive or basal glucose uptake, has a very broad substrate specificity, and can transport a wide range of aldoses including both pentoses and hexoses
6	GRM5	Glutamate metabotropic receptor 5	Ligand binding causes a conformation change that triggers signaling via guanine nucleotide-binding proteins (G proteins) and modulates the activity of down-stream effectors
7	PLCB4	Phospholipase C beta 4	The production of the second messenger molecules diacylglycerol (DAG) and inositol 1,4,5-trisphosphate (IP3) is mediated by activated phosphatidylinositol-specific phospholipase C enzymes
8	FGFR1	Fibroblast growth factor receptor 1	Tyrosine-protein kinase that acts as cell-surface receptor for fibroblast growth factors and plays an essential role in the regulation of embryonic development, cell proliferation, differentiation, and migration
9	UBE2V2	Ubiquitin conjugating enzyme E2 V2	Plays a role in the control of progress through the cell cycle and differentiation, plays a role in the error-free DNA repair pathway, and contributes to the survival of cells after DNA damage
10	TNFRSF10B	TNF receptor superfamily member 10b	Promotes the activation of NF-kappa-B. Essential for ER stress-induced apoptosis

**Table 5:** The demographic data and the expression status of CDKN2A.

			CDKN2A	
			Low (%)	High (%)
Sex	Male	181	119 (56.1%)	62 (29.2%)
	Female	31	0 (0.0%)	31 (14.6%)

Age	<65 years	100	64 (30.2%)	36 (17.0%)
	≥65 years	112	55 (25.9%)	57 (26.9%)

Tumor location	LCOAD	106	59 (27.8%)	47 (22.2%)
	RCOAD	106	60 (28.3%)	46 (21.7%)

Overall survival	<60 months	122	57 (26.9%)	65 (30.7%)
	≥60 months	90	62 (29.2%)	28 (13.2%)

## Data Availability

The datasets used and/or analyzed during the current study are available from the corresponding author on reasonable request.

## Abbreviations

COAD: Colon adenocarcinoma
LCOAD: Left colon adenocarcinoma
RCOAD: Right colon adenocarcinoma
GEO: Gene expression omnibus
DEGs: Differentially expressed genes
GO: Gene Ontology
KEGG: Kyoto Encyclopedia of Genes and Genomes
PPI: Protein-protein interaction
GEPIA: Gene expression profiling interactive analysis
BP: Biological process
CC: Cellular component
MF: Molecular function
BiNGO: Biological networks gene oncology tool
OS: Overall survival
CDKN2A: Cyclin-dependent kinase inhibitor 2A
MDM2: MDM2 proto-oncogene
SLC2A1: Solute carrier family 2 member 1.
